# Adhering to Healthy Dietary Patterns Prevents Cognitive Decline of Older Adults with Sarcopenia: The Mr. OS and Ms. OS Study

**DOI:** 10.3390/nu17193070

**Published:** 2025-09-26

**Authors:** Yichen Jin, Gianna Lai, Shuyi Li, Jenny Lee, Vicky Chan, Zhihui Lu, Jason Leung, Kingson Lai, Kuen Lam, Tung Wai Auyeung, Timothy Kwok, Kwok Tai Chui, Jean Woo, Kenneth Ka-hei Lo

**Affiliations:** 1 Jockey Club School of Public Health and Primary Care, The Chinese University of Hong Kong, Shatin, New Territories, Hong Kong SAR, China; olivia.jin@connect.hku.hk; 2 Department of Food Science and Nutrition, The Hong Kong Polytechnic University, 11 Yuk Choi Road, Hung Hom, Kowloon, Hong Kong SAR, China; zhiyang.lai@connect.polyu.hk (G.L.); waiki-vicky.chan@connect.polyu.hk (V.C.); 3 Department of Medicine and Therapeutics, The Chinese University of Hong Kong, Shatin, New Territories, Hong Kong SAR, China; 4 Jockey Club Institute of Ageing, The Chinese University of Hong Kong, Shatin, New Territories, Hong Kong SAR, China; 5 School of Nursing, The Hong Kong Polytechnic University, 11 Yuk Choi Road, Hung Hom, Kowloon, Hong Kong SAR, China; 6 Jockey Club Centre for Osteoporosis Care and Control, The Chinese University of Hong Kong, Shatin, New Territories, Hong Kong SAR, China; jason-leung@cuhk.edu.hk; 7 Department of Medicine and Geriatrics, Tai Po Hospital, 9 Chuen On Rd, Tai Po, New Territories, Hong Kong SAR, China; 8 Department of Medicine and Geriatrics, Shatin Hospital, 33 A Kung Kok Street, Shatin, New Territories, Hong Kong SAR, China; 9 School of Science and Technology, Hong Kong Metropolitan University, Hong Kong SAR, China; jktchui@hkmu.edu.hk

**Keywords:** sarcopenia, cognitive decline, dietary patterns, protein intake, older adults

## Abstract

**Background**: The progression of cognitive decline is accelerated in older adults with sarcopenia, but the protective dietary factors have remained uncertain. **Objective**: This study aims to investigate the association between dietary factors and cognitive decline in older adults, and to explore the potential mediating effects of sarcopenic components. **Methods**: Data from the Mr. OS and Ms. OS cohort study in Hong Kong (*N* = 3146, aged ≥65 years) were used. Cognitive function was assessed based on the Mini-Mental State Examination (MMSE). Sarcopenic status was assessed according to the Asian Working Group for Sarcopenia 2019 updated consensus. Dietary protein intake and adherence to dietary patterns were assessed using a food frequency questionnaire. Linear regression was used to examine the associations between dietary factors and MMSE scores. Mediation analysis was conducted to identify the possible mediators in the diet–cognition associations. **Results**: Sarcopenia and its components were associated with baseline MMSE and MMSE changes. Positive associations were observed for plant protein intake (β = 0.79, 95% CI 0.24–1.35) and dietary patterns such as the Dietary Approaches to Stop Hypertension (DASH) diet (β = 0.14, 95% CI 0.02–0.26) and diets with lower Dietary Inflammatory Index (DII) scores (β = −0.18, 95% CI −0.26–−0.09) with better MMSE outcomes. Protective effects were more profound in participants with sarcopenia/severe sarcopenia. The effects of the DASH diet and DII were more profound in female participants, while higher adherence to the Mediterranean–DASH Intervention for Neurodegenerative Delay (MIND) diet was associated with an increment in MMSE score in male participants with sarcopenia. Handgrip strength and physical performance are significant mediators in the diet–cognition associations. **Conclusions**: The protective effects of healthy dietary patterns were beneficial, especially for participants with sarcopenia, while handgrip strength and walking speed potentially mediated the associations.

## 1. Introduction

Dementia is a significant global health issue, with millions of new cases diagnosed each year, leading to a high economic cost and a considerable impact on quality of life. The number of people living with dementia could nearly triple from approximately 57 million in 2019 to 152 million by 2050 [1]. Cognitive decline is a preclinical stage of dementia, which can be reverted to normal cognition [2]. Therefore, identifying high-risk populations and modifiable factors is important. On the one hand, biological aging is a major contributor to cognitive decline; the role of sarcopenia in accelerating cognitive decline has been recognized in recent years. Sarcopenia, as characterized by the age-related loss of skeletal muscle mass and strength [3], affects 12.9% of the world’s aging population [4]. From a physiological perspective, sarcopenia-related muscle loss might accelerate cognitive decline via changes in myokine secretion, proinflammatory cytokine production, and poor vascular homeostasis [5]. From an epidemiological perspective, a meta-analysis of 18,788 participants worldwide showed that the likelihood of developing cognitive impairment was significantly higher in individuals with sarcopenia than in those without (odds ratio: 1.75), and there are significantly lower Mini-Mental State Examination (MMSE) scores among people with sarcopenia than among their counterparts (mean difference = −2.23) [6]. Other components of sarcopenia, such as low gait speed and grip strength, also predict the risk of cognitive decline and dementia [7]. It is worth noting that low walking speed is not only an important phenotype of sarcopenia but also the core diagnostic feature of motoric cognitive risk syndrome (MCR). Longitudinal studies have shown that both sarcopenia and MCR are associated with cognitive decline within 3–4 years [8,9,10]. In terms of sarcopenia management, previous literature suggests the importance of nutrition in bone health via bone mineralization; recent evidence has suggested the benefit of nutrition being extended to sarcopenia, an interconnected condition [11,12].

In view of the linkage between sarcopenia and cognitive decline, it is important to identify lifestyle factors that may exacerbate or mitigate the progression of cognitive decline for individuals with sarcopenia. The potential of nutritional interventions has been recognized via their role in reducing oxidative stress and inflammation, as well as improving vascular health and neuronal cell signaling [13,14]. However, research evidence from nutritional interventions is limited and mainly focused on individual dietary supplement trials [15]. Moreover, dietary patterns have gained increased attention, as they account for the combination of and synergy among various food types, with stronger practical value [16]. Accumulating evidence suggests that some dietary patterns, such as the Dietary Approaches to Stop Hypertension (DASH) diet, the Mediterranean diet, the Mediterranean–Dietary Approaches to Stop Hypertension Intervention for Neurodegenerative Delay (MIND) diet, and the anti-inflammatory diet may have a protective effect on cognitive impairment in the general population [17,18]. While details may vary, there are shared features in healthy dietary patterns, e.g., the consumption of green leafy vegetables, nuts, and fish, which showed independent positive associations with cognitive function [19]. The association between fish consumption and cognitive function also accords with previous studies that focused on assessing dietary protein intake [20,21]. While the current dietary recommendation for people with sarcopenia focuses on adequate protein intake to improve muscle health, there is potential to extend the benefit to cognitive outcomes, as well as identify dietary patterns that can improve cognitive health.

However, there has been no prospective cohort analysis on dietary factors related to the changes in cognitive function in older adults with sarcopenia. In this study, we used data from the Mr. OS and Ms. OS cohort in Hong Kong. This cohort was established between 2001 and 2003, recruiting about 4000 community-dwelling older adults aged 65 years and above. It was initially aimed to investigate osteoporosis, sarcopenia, and other aging-related health outcomes, and through multiple follow-ups has become an important resource for geriatric research. To fill the research gap, we set out three objectives. First, we assessed the relationship between sarcopenia and its components with cognitive decline in older adults. Second, we examined the association between protein intake, dietary patterns, and cognitive decline in general older adults and a sub-cohort of participants with sarcopenia, and also conducted analysis by sex. Third, we explored the sarcopenic components as potential mediators in the diet–cognition associations. We hypothesized that higher protein intake and better adherence to healthy dietary patterns are linked to slower cognitive decline as measured by the MMSE, with part of this relationship mediated by sarcopenia-related factors such as muscle strength and physical performance.

## 2. Materials and Methods

### 2.1. Study Design and Population

The Mr. OS and Ms. OS cohort study recruited 2000 men and 2000 women aged 65 or above living in the Hong Kong community between 2001 and 2003 [22]. A stratified sample was used to equally assign one-third of the participants into three age groups: 65–69, 70–74, and ≥75. A total of 3991 participants without missing data on exposure, outcome, and covariates at baseline were included in the present study. Of these, 3146 participants with completed MMSE scores at baseline and in the fourth year were included in the analysis of MMSE change.

This study was conducted following the guidelines set out in the Declaration of Helsinki and was approved by the Clinical Research Ethics Committee of The Chinese University of Hong Kong (CRE: 2003.102). Written informed consent was obtained from all participants.

### 2.2. Cognitive Outcomes

Cognitive function was assessed based on the Mini-Mental State Examination (MMSE) [23] at baseline and reassessed in the fourth year. MMSE assessment includes several domains: orientation, immediate and short-term recall, attention and calculation, word finding, figure construction, reading and writing skills, and ability to follow a three-step command. The total scores range from 0 to 30. MMSE change was calculated as the year-4 MMSE score minus the baseline MMSE score.

### 2.3. Sarcopenia Assessment

Sarcopenia was assessed at baseline according to the Asian Working Group for Sarcopenia (AWGS) 2019 updated consensus [24]. Participants were classified into four groups: non-sarcopenia (healthy); possible sarcopenia (low muscle strength, defined as grip strength <28 kg in men and <18 kg in women, or low physical performance, defined as 6 m walk <1.0 m/s or 5-time chair stand ≥12 s); sarcopenia (low appendicular skeletal muscle mass (ASM) index (ASM/height^2^) according to dual-energy X-ray absorptiometry (DXA), <7.0 kg/m^2^ in men and <5.4 kg/m^2^ in women, with either low muscle strength or low physical performance); and severe sarcopenia (low ASM with low muscle strength and low physical performance).

### 2.4. Dietary Assessment

Dietary intake was assessed at baseline using a validated food frequency questionnaire (FFQ) with 280 food items, which was previously validated against 24 h dietary recalls in the older Hong Kong population [25]. The FFQ was administered through face-to-face interviews by trained staff. Participants were asked to report the frequency and portion size for each food item consumed over the past 12 months. A food photo catalog with standard portion sizes was provided to assist in portion estimation [26]. The calculation of daily protein intake used the food tables derived from McCance and Widdowson [27] and the Chinese Medical Sciences Institute [28]. Energy-adjusted intakes were computed using the residual method [29]. Total protein intake was calculated as the sum of animal and plant protein [30]. Animal protein sources included red meat, processed meat, poultry, fish and shellfish, eggs, milk products, animal giblets, and others [30]. Plant protein sources included cereals, vegetables, fruits, soybeans, nuts and seeds, and others [30].

The Diet Quality Index—International (DQI-I) assessment includes four aspects of the diet with subcomponents: variety, adequacy, moderation, and overall balance. The overall score ranges from 0 to 100. Due to insufficient information to calculate the category of empty-calorie foods under the ‘moderation’ aspect in this study, the scoring range for moderation was adjusted to 0–24 instead of the usual 0–30, and the total score of the DQI-I ranged from 0 to 94 instead of 0 to 100, with a high score indicating high quality.

The DASH score was calculated based on the method developed by Mellen et al. [31]. The DASH target intakes included nine nutrients (i.e., total fat, saturated fat, protein, fiber, cholesterol, calcium, magnesium, potassium, and sodium). One score would be given when achieving each target intake. A score of 0.5 was assigned when achieving a nutrient target between the DASH target and the nutrient content of the control group’s diet in the DASH trial. The total score ranged from 0 to 9, with a higher score representing greater adherence.

The MIND score was calculated based on 15 food groups associated with the prevention of cognitive impairment: ten beneficial food groups (green leafy vegetables, other vegetables, nuts, berries, beans, whole grains, seafood, poultry, olive oil, and wine) and five unhealthy food groups (red meats, butter and stick margarine, cheese, pastries and sweets, and fried/fast food). Each food group was given a score of 0, 0.5, or 1 based on the frequency and proportion of consumption [32]. However, this study lacked sufficient information on the consumption of olive oil and the frequency of consuming fish (not fried), beans, poultry, red meat and products, and fast-fried foods, so the maximum MIND score in our study was 9 instead of 15.

The Mediterranean Diet Score (MDS) was calculated using the methods developed by Trichopoulou et al. [33]. One score was given when the consumption of beneficial components (vegetables, legumes, fruits and nuts, cereal, and fish) was at or above the sex-specific median, and the consumption of detrimental components was below the median cutoff. A value of 1 was assigned if daily consumption of ethanol was between 10 and 50 g for men or 5 and 25 g for women.

The dietary inflammatory index (DII) was calculated based on 30 literature-derived food parameters related to chronic inflammation [34]. The DII score was calculated as the sum of the standardized scores of 30 food parameters. A higher and positive DII score indicates a more proinflammatory diet, while a lower and negative one indicates a more anti-inflammatory diet.

### 2.5. Justification for Inclusion of Dietary Patterns

There is growing evidence supporting the use of holistic dietary patterns rather than individual nutrients or food groups in promoting cognitive health among older adults [35]. We included five established dietary pattern scores (MIND, DASH, MDS, DQI-I, and DII) because of their known relevance to cognitive and brain health [36]. We also aimed to examine whether these patterns may influence cognition indirectly through sarcopenic components.

The MIND diet has been linked to slower cognitive decline and a reduced risk of dementia, with a focus on plant-based foods such as green leafy vegetables, berries, whole grains, and nuts [19]. The DASH and MDS scores were selected due to their well-documented roles in supporting cardiovascular and brain health [36]. The DQI-I reflects overall dietary quality and has been validated among East Asian populations, including older Hong Kong adults [37]. The DII captures the inflammatory potential of the diet and has been associated with neuroinflammation and cognitive decline [38,39].

All five dietary pattern scores have been applied in the Mr. and Ms. OS cohort in Hong Kong [37,40].

### 2.6. High-Sensitivity C-Reactive Protein (hs-CRP)

To measure the levels of chronic inflammation, fasting serum samples were collected from the participants at baseline and stored at −80 °C. The serum hs-CRP level was measured using a commercially available enzyme-linked immunosorbent assay (Vitros Fusion 5,1, Vitros Chemistry Products, Raritan, NJ, USA). The intra-assay and inter-assay coefficients of variation (CVs) were 1.1–1.4% and 3.7–6.2%, respectively.

### 2.7. Covariates

A standardized, structured interview was performed to collect baseline information on age, sex, body mass index, systolic blood pressure, education level, smoking habit (never, former, or current smoker), alcohol use (≥12 alcoholic drinks in the past year), and self-reported medical history (diabetes, stroke, heart attack, angina, congestive heart failure, or cancer). Physical activity level was assessed using the Physical Activity Scale for the Elderly (PASE) score, with a higher score indicating greater intensity of physical activity [41].

### 2.8. Statistical Analysis

Baseline characteristics were stratified by sarcopenia status using means with standard deviations for continuous variables and frequencies with percentages for categorical variables. One-way ANOVA tests and chi-square tests were used for continuous variables and categorical variables, respectively. In this study, MMSE scores and their changes, protein intake (g/kg/day), dietary pattern scores (DQI-I, DASH, MIND, Mediterranean, DII), BMI, SBP, PASE score, handgrip strength, walking speed, chair stand time, and muscle mass were treated as continuous variables. Sex, education level, smoking status, alcohol drinking, and medical history (diabetes, stroke, heart attack, angina, heart failure, cancer) were treated as categorical variables. Sarcopenia status (healthy, probable, sarcopenia/severe sarcopenia) and its diagnostic components (low handgrip strength, low physical performance, low muscle mass) were also considered categorical. Dietary exposures were analyzed both as continuous variables and as quartile categories to test linear trends and distributional effects. Linear regression was used to estimate the coefficient (β) and 95% CIs for the association between sarcopenia and MMSE scores (baseline MMSE and MMSE change), in addition to the association between dietary factors and MMSE score among all participants and a sub-cohort of participants with sarcopenia/severe sarcopenia. To enhance statistical power, we combined participants with sarcopenia and severe sarcopenia into a single group for analysis. The sex-stratified analyses were also conducted for the diet–cognition associations. The models were adjusted for sex, age, dietary energy, body mass index, physical activity, systolic blood pressure, medical history, smoking habit, alcohol drinking, and education level. We additionally performed sensitivity analyses excluding BMI and PASE score, as these covariates may lie on the causal pathway between diet and cognition.

Mediation analysis was conducted to explore the potential mediation effect of sarcopenia-related mediators (handgrip strength, walking speed, time to complete 5 stands, and muscle mass) and hs-CRP in the association between dietary factors and cognitive outcomes among all participants and participants with sarcopenia/severe sarcopenia. We integrated two regression models, one to regress the cognitive outcomes on the exposure and the mediator, and a linear model to regress the mediator on the exposure, with adjustment for covariates [42,43]. The mediation analysis was performed using the “mediation” package in R. The proportion mediated was estimated on the coefficient scale, and the 95% CIs were obtained using bootstrapping. All analyses were carried out with SPSS statistical software version 27.0.1 (SPSS, Inc., Chicago, IL, USA) and R 4.3.2 software. We assumed that dietary factors at baseline influence sarcopenia-related components, which in turn affect the cognitive outcomes at year 4. A 2-sided *p* value of less than 0.05 is considered statistically significant.

## 3. Results

### 3.1. Characteristics of OS Study Participants

After excluding 9 participants with missing data on dietary factors or covariates and 845 participants with no year-4 MMSE score records, a total of 3146 older adults were included in the analysis. The baseline characteristics of these participants are presented by sarcopenia status in Table 1. A total of 1572 (50.0%) and 576 (18.3%) participants were diagnosed with probable sarcopenia and sarcopenia/severe sarcopenia, respectively. Compared with healthy participants, older adults with sarcopenia/severe sarcopenia tended to be males; be of older age; be less likely to have post-secondary education; have lower PASE scores, BMI, and systolic blood pressure; be more likely to be current smokers; have a stroke history; have a higher protein intake (total protein, animal protein, and plant protein); have lower scores for the DQI-I, DASH, MIND, and Mediterranean diet; and have higher DII scores. Supplementary Table S1 shows the sex-specific levels of dietary intake in all participants and participants with sarcopenia/severe sarcopenia. Compared with all women, men had higher energy and protein intakes, and lower DQI-I, DASH diet, MIND diet, and DII scores. However, in the participants with sarcopenia/severe sarcopenia, there was no significant difference in plant protein intake compared to women.

### 3.2. Sarcopenia and Cognitive Outcomes

Table 2 shows the associations between baseline sarcopenia status, diagnosis components, and MMSE scores. Compared with the non-sarcopenia group, probable sarcopenia was significantly associated with a greater reduction in MMSE scores (β = −0.51, 95% CI = −0.76 to −0.26), and sarcopenia/severe sarcopenia was related to a greater reduction in MMSE scores (β = −0.52, 95% CI = −0.85 to −0.19).

For three diagnosis components of sarcopenia, low handgrip strength was associated with a greater reduction in MMSE scores (β = −0.61, 95% CI = −0.97 to −0.25). Per 1-unit higher handgrip strength was associated with a higher baseline MMSE (β = 0.03, 95% CI = 0.01 to 0.05) and increment in MMSE scores (β = 0.04, 95% CI = 0.02 to 0.06). Low physical performance was associated with a greater reduction in MMSE scores (β = −0.46, 95% CI = −0.69 to −0.23). Per 1-unit higher walking speed was associated with a higher baseline MMSE (β = 0.83, 95% CI = 0.30 to 1.36) and MMSE score increment (β = 0.71, 95% CI = 0.17 to 1.24). Per 1-unit longer time to complete five stands was associated with a greater reduction in MMSE scores (β = −0.04, 95% CI = −0.07 to −0.02). Per 1-unit greater muscle mass was associated with lower baseline MMSE scores (β = −0.25, 95% CI = −0.47 to −0.02).

### 3.3. Protein Intake, Dietary Patterns, and Cognitive Outcomes

**Figure 1:**
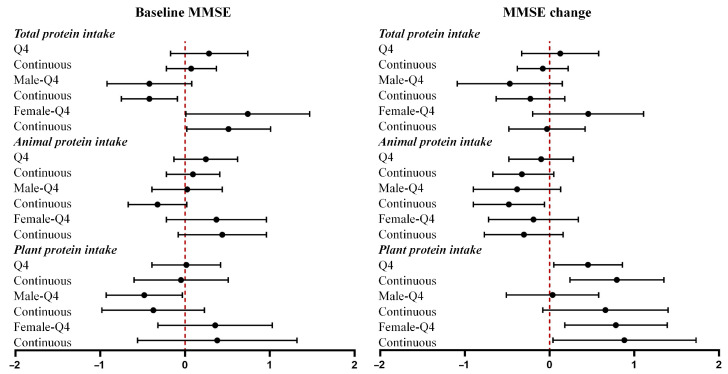
Associations of protein intake with baseline MMSE and MMSE change. Forest plots showing β coefficients (95% CI) for associations of total, animal, and plant protein intake with baseline MMSE and MMSE change, stratified by sex. Q4 = highest quartile vs. Q1 (reference). Continuous = per 1-unit increment.

**Figure 2:**
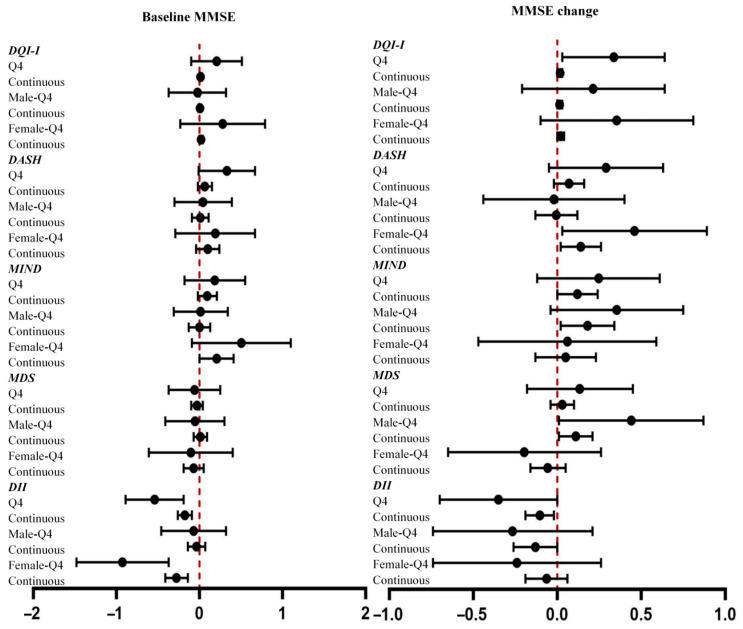
Associations of dietary patterns with baseline MMSE and MMSE change. Forest plots showing β coefficients (95% CI) for associations of dietary pattern scores (DQI-I, DASH, MIND, MDS, and DII) with baseline MMSE and MMSE change, stratified by sex. Q4 = highest quartile vs. Q1 (reference). Continuous = per 1-unit increment.

The associations between protein intake, dietary patterns, and MMSE scores among all participants are summarized in Figure 1 and Figure 2. Full regression results are provided in Supplementary Tables S3 and S4. Per 1-unit increment in total protein intake was associated with a lower baseline MMSE in males (β = −0.42, 95% CI = −0.75 to −0.09) and higher baseline MMSE in females (β = 0.51, 95% CI = 0.02 to 1.01). Per 1-unit increment in animal protein intake was associated with a greater reduction in MMSE scores in all participants (β = −0.36, 95% CI = −0.67 to −0.05) and in males (β = −0.48, 95% CI = −0.90 to −0.06). Per 1-unit increment in plant protein intake was associated with an increment in MMSE scores in all participants (β = 0.79, 95% CI = 0.24 to 1.35) and in females (β = 0.88, 95% CI = 0.04 to 1.73).

Per 1-unit increment in DQI-I was associated with a higher baseline MMSE (β = 0.01, 95% CI = 0.00 to 0.03). Per 1-unit increment in DQI-I was associated with an increment in MMSE scores in all participants (β = 0.02, 95% CI = 0.00 to 0.03) and all females (β = 0.02, 95% CI = 0.00 to 0.04). The highest quartile of the DQI was associated with an increment in MMSE scores in all participants (β = 0.34, 95% CI = 0.03 to 0.64). The highest quartile of the DASH diet (β = 0.46, 95% CI = 0.03 to 0.89) and per 1-unit increment in the DASH diet score (β = 0.14, 95% CI = 0.02 to 0.26) were associated with an increment in MMSE scores in females. Per 1-unit increment in the MIND diet score was associated with a higher baseline MMSE in females (β = 0.21, 95% CI = 0.00 to 0.41) and increment in MMSE scores in males (β = 0.18, 95% CI = 0.02 to 0.34). The highest quartile of the MDS (β = 0.44, 95% CI = 0.01 to 0.87) and per 1-unit increment in the MDS (β = 0.11, 95% CI = 0.01 to 0.21) were associated with an increment in MMSE scores in males. ^†^The sex × exposure interaction (tested on the continuous term) is significant at *p* < 0.05. The sex-stratified estimates are exploratory.

The highest quartile of the DII score and per 1-unit increment in DII score were associated with baseline MMSE in all participants (Q4: β = −0.54, 95% CI = −0.89 to −0.19; continuous: β = −0.18, 95% CI = −0.26 to −0.09) and in females (Q4: β = −0.93, 95% CI = −1.48 to −0.37; continuous: β = −0.28, 95% CI = −0.41 to −0.14). The highest quartile of the DII score and per 1-unit increment in DII score were associated with MMSE reductions in all participants (Q4: β = −0.35, 95% CI = −0.70 to −0.00; continuous: β = −0.10, 95% CI = −0.19 to −0.02). Per 1-unit increment in DII score was also associated with MMSE reduction in males (Q4: β = −0.35, 95% CI = −0.70 to −0.00; continuous: β = −0.10, 95% CI = −0.19 to −0.02). The results of the regression analysis among participants with sarcopenia/severe sarcopenia are presented in Supplementary Tables S5 and S6. Sensitivity analyses excluding BMI and PASE score yielded results largely consistent with the main findings (Supplementary Table S7).

### 3.4. Mediation Analysis

**Figure 3:**
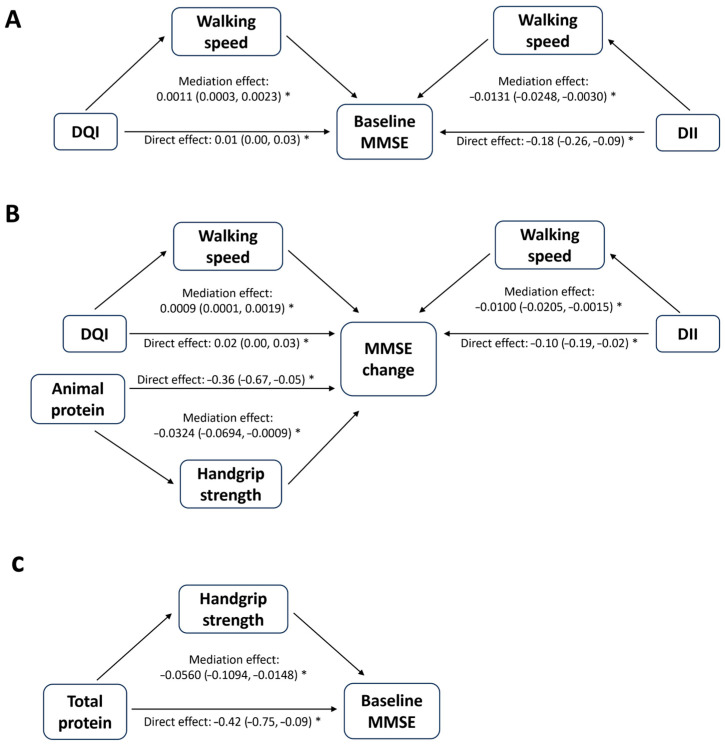
Model diagram for significant mediating effects of sarcopenia-related components on the association of dietary factors with MMSE scores. Notes: (**A**) Mediating effects of sarcopenia-related components on the association of dietary factors with baseline MMSE scores in all participants. (**B**) Mediating effects of sarcopenia-related components on the association of dietary factors with MMSE change in all participants. (**C**) Mediating effects of sarcopenia-related components on the association of dietary factors with baseline MMSE scores in all male participants. Abbreviations: DQI-I, Diet Quality Index—International; MMSE, Mini-Mental State Examination; DII, dietary inflammation index. * *p* < 0.05.

Potential mediators that were significantly associated with exposures, as well as with baseline MMSE/MMSE change, were further investigated in mediation analysis (Table 3). Supplementary Table S8 presents the results for the significant associations between exposures (dietary factors) and mediators (sarcopenia-related components). For all participants, handgrip strength, walking speed, and time to complete five stands are significant mediators in the association between dietary factors and MMSE scores. For the overall effect of the DQI-I and DII on baseline MMSE scores, walking speed mediated 8.04% and 7.43% of the total effects, respectively. Time to complete five stands mediated 9.31% and 37.22% of the total effect of the DQI-I and DII on MMSE change, respectively. Handgrip strength mediated 8.05% of the total effect of animal protein on MMSE change. For all males, the associations of total protein with baseline MMSE were mediated by handgrip strength (13.36%). The associations of the DII with MMSE change were mediated by time to complete five stands (15.05%). The model diagram for mediation analysis is shown in Figure 3.

## 4. Discussion

To the best of our knowledge, this is one of the first prospective cohort studies to investigate the associations between dietary factors and cognitive decline in older adults with and without sarcopenia, and to further explore the diet–muscle–cognition triad through mediation analysis. Our findings demonstrated the adverse effect of sarcopenia and two sarcopenic components (i.e., poor handgrip strength and low physical performance) on cognitive function, which was consistent with previous studies [44,45,46,47]. The association between sarcopenia and worse cognitive health also indicated the importance of identifying protective dietary factors, especially in this vulnerable population. Our mediation analyses further showed that two sarcopenic components, handgrip strength and walking speed, exhibited significant mediating effects in the diet–cognition associations.

In our study, the protective effect of plant protein intake on cognitive function was found among all participants, but the effect was debatable. Consistent with our findings on the more pronounced effect of plant protein on the increment in MMSE scores, previous studies have also highlighted the cognitive benefits of plant protein over animal protein [20,48]. A study using two US cohorts reported that substituting every 5% of energy from animal protein with plant protein was associated with 16% lower odds of subjective cognitive decline [20]. The effect of protein intake on cognitive function may be explained by the impact of dietary proteins on the specific amino acids which are essential for synthesizing neurotransmitters [49]. Research has indicated that tryptophan, which is a necessary protein for brain health and cognitive processes, may be more efficiently utilized in synthesizing neurotransmitters when derived from plant sources compared to animal sources [50]. Our findings together with the previous evidence suggested that the neuroprotective effects of plant proteins could potentially make them a superior protein source for mitigating cognitive decline. In the sex-stratified analysis, the impact of protein intake was notably greater in women, consistent with previous findings [51,52]. Some studies have suggested that women may need more protein intake than men due to higher levels of protein oxidation [53]. However, the baseline data (Supplementary Table S1) in our cohort showed significantly lower protein consumption among women than men, potentially resulting in a heightened effect. Current evidence on the associations between protein intake and cognitive function is inconclusive [20,51,54,55], which may be attributed to differences in study design, the length of follow-up, and the characteristics of the participants, and there remains a lack of evidence among people with sarcopenia.

Our findings on dietary patterns in all participants were generally in line with the results from the previous literature. Many studies have been conducted to explore the association of dietary patterns with cognitive decline. A systematic review [18] suggested an overall protective effect of the Mediterranean, MIND, DASH, and anti-inflammatory diets on cognitive function in older adults. Common features exist in the three dietary patterns (Mediterranean, MIND, and DASH diets), such as emphasizing the high consumption of grains, fresh fruit, vegetables, and nuts [18]. Together with the anti-inflammatory diet with a low DII score, abundant bioactive compounds such as carotenoids, polyphenols, and antioxidant vitamins are consumed [17]. These nutrients have been reported to be antioxidants and anti-inflammatory agents and have neuroprotective properties [56]. When compared to previous literature, our findings demonstrated that the benefits of these dietary patterns in slowing cognitive decline are more profound in older adults with sarcopenia. Our results also revealed sex differences in the effects of specific dietary factors on cognitive decline. Higher adherence to the DASH diet was associated with an increment in MMSE score in female participants with sarcopenia, while higher adherence to the MIND diet was associated with an increment in MMSE score in male participants with sarcopenia. The protective effect of the DASH diet in women was also reported to lower the prevalence of prospective subjective cognitive complaints in women [57]. A Japanese study using the MMSE for cognition assessment found a negative association between soy food, isoflavones, and cognitive decline specifically in elderly women, rather than men [58]. Additionally, calcium intake and dairy products were observed to be inversely associated with cognitive decline [59,60], while inadequate calcium intake is frequently noted among elderly women. This may offer a potential explanation for the observed effects of the DASH diet in females, as the DASH diet emphasizes the intake of soy food and low-fat dairy products. Meanwhile, the sex difference in the cognitive effect of MIND diet adherence remains inconclusive. A study using data from UK Biobank observed an inverse association between the MIND diet and dementia only in women [61], while an insignificant association in females was reported in other studies [62]. The risk effect of the DII on cognitive decline was only found in women (in both all women and women with sarcopenia) in our study, which was consistent with previous studies showing that inflammatory markers such as CRP had a statistically significant negative association with cognitive functioning for females but not males [63]. More future studies may explore the diet–cognition associations according to sex and sarcopenic status, and the underlying mechanisms.

In the mediation analysis, our results showed that the associations between several dietary patterns and cognitive outcomes were significantly mediated by handgrip strength and physical performance (walking speed and time to complete five stands) in all participants. Previous research suggested that sarcopenia was linked to cognitive decline via proinflammatory cytokine production caused by the reduction in myokine secretion under low skeletal muscle mass [5]. Interestingly, a mediating effect of low muscle mass between the associations of healthy dietary patterns and cognitive outcomes was not observed. Both the frontal lobe and hippocampus are vulnerable to neurodegenerative changes, and these changes have been linked to decreased gait speed and subjective cognitive complaints [64,65]. In other words, physical performance may serve as the early marker of cognitive impairment, echoing the concept of motoric cognitive risk syndrome [66].

Previous literature has reported the roles of healthy dietary patterns in improvements in handgrip strength [67] and gait speed [68,69]. In our study, we revealed that a higher DQI-I and DII and protein intake could influence cognitive function via muscle strength and physical performance. Additionally, it is worth noting that our results indicated that protein intake was related to lower MMSE scores, which might be mediated by reduced handgrip strength. The association between higher protein consumption and low handgrip strength could potentially be attributed to participants with sarcopenia (low handgrip strength) consciously increasing their protein intake, which is observed in our cohort (Table 1). However, participants with sarcopenia had lower adherence to several healthy dietary patterns compared with the healthy participants, which might explain the associations between dietary patterns and cognitions in this sub-cohort. Several common features of healthy dietary patterns, such as the emphasis on vegetables, berries, whole grains, fish, olives, and nuts, have been reported to be beneficial to physical function and grip strength [70,71]. The rich contents of inorganic nitrate, polyphenols, and *n*-3 fatty acids from these food components have been shown to support muscle strength and enhance physical function [70,71]. Our findings suggest that greater adherence to healthy dietary patterns, regardless of their specific types, may have beneficial effects on cognition for older adults in general, as well as for those with sarcopenia.

The choice of a 4-year follow-up period in this study is reasonable, because previous studies have shown that both sarcopenia and MCR can predict cognitive decline or dementia risk within about 3 years of follow-up. Therefore, evaluating the protective effects of dietary factors within this time window has direct clinical and public health significance. The present study has several strengths and implications. We conducted objective physical measurements for sarcopenia diagnosis, as well as the dietary intake measurement using a validated FFQ. With the prospective study design and large cohort with few missing data, our results provide evidence for the association between diet, sarcopenia, and cognitive outcomes. The single assessment of dietary intake at baseline may not have reflected the long-term dietary intakes of the participants, which may have been influenced by the diagnosis and medication of diseases during follow-up. However, we contend that dietary changes over four years may not be substantial, as several nutrition cohorts (e.g., Nurse’s Health Study, Health Professional Follow-up Study, Women’s Health Initiative) have conducted cumulative dietary assessments in three to four years. In addition, the temporal assumption is inherent in the mediation model and represents a limitation, as dietary intake was only measured at baseline. In addition, as the data were collected about two decades ago, dietary habits and lifestyle may differ from those of today’s older population, and the findings should be interpreted with caution when generalized to current cohorts. Moreover, some studies suggested a better validity of using the MoCA rather than the MMSE for mild cognitive impairment assessment [72], but only MMSE data are accessible for our cohort. Residual confounding factors such as stress and depressive symptoms would exist, although the models have been carefully adjusted for other potential confounders. For future research, there remains a need for longitudinal cohort studies to further track the long-term associations between dietary intake and the progression of cognitive decline in populations with sarcopenia, in addition to the effects of nutrition interventions on muscle and cognitive functions.

## 5. Conclusions

In conclusion, protein intake and dietary patterns were significantly associated with cognitive outcomes in community-dwelling older Hong Kong adults, and such associations were more profound in participants with sarcopenia/severe sarcopenia, potentially mediated by handgrip strength and walking speed.

These findings suggest that promoting a healthy diet is an evidence-based public health strategy, which may help delay cognitive and physical function decline in older adults, especially in those with sarcopenia, a population at high risk of cognitive decline. Future studies should combine evidence from long-term follow-up and explore integrated interventions pertaining to nutrition and exercise to further improve cognitive and physical health in older populations.

## Figures and Tables

**Table 1:** Baseline characteristics of participants in Mr OS and Ms OS study according to sarcopenic status (*N* = 3146).

	Healthy (*N* = 998)	Probable Sarcopenia (*N* = 1572)	Sarcopenia/Severe Sarcopenia (*N* = 576)	*p* Value
	Mean (SD)/*N* (%)			
Sex (female)	437 (43.8)	966 (61.5)	179 (31.1)	<0.001
Age (years)	70.31 ± 4.16	72.41 ± 4.83	73.27 ± 5.41	<0.001
Post-secondary education	163 (16.3)	108 (6.9)	58 (10.1)	<0.001
Physical activity (PASE score)	100.51 ± 46.53	91.76 ± 41.42	87.37 ± 41.58	<0.001
Smoking habit				<0.001
Former smoker	271 (27.2)	421 (26.8)	224 (38.9)	
Current smoker	73 (7.3)	57 (3.6)	66 (11.5)	
Drank > 12 alcoholic drinks in the past year	158 (15.8)	177 (11.3)	90 (15.6)	0.001
Dietary energy (kcal)	1916.58 ± 600.98	1790.86 ± 572.29	1917.66 ± 575.86	<0.001
Body mass index (kg/m^2^)	23.53 ± 3.11	24.90 ± 2.90	20.96 ± 2.34	<0.001
Systolic blood pressure	141.78 ± 19.16	143.34 ± 18.45	139.81 ± 18.85	<0.001
History of diabetes	131 (13.1)	252 (16.0)	59 (10.2)	0.002
History of stroke	24 (2.4)	69 (4.4)	31 (5.4)	0.006
History of heart attack	84 (8.4)	161 (10.2)	48 (8.3)	0.201
History of angina	76 (7.6)	143 (9.1)	42 (7.3)	0.260
History of congestive heart failure	27 (2.7)	71 (4.5)	21 (3.6)	0.063
History of cancer	33 (3.3)	77 (4.9)	19 (3.3)	0.079
Baseline MMSE	26.50 (3.12)	25.39 (3.68)	26.36 (3.05)	<0.001
MMSE change	0.38 (3.12)	0.17 (3.41)	−0.38 (3.71)	<0.001
Protein (g) per day/kg	1.38 ± 0.61	1.24 ± 0.52	1.51 ± 0.65	<0.001
Animal protein (g) per day/kg	0.77 ± 0.46	0.70 ± 0.40	0.87 ± 0.50	<0.001
Plant protein (g) per day/kg	0.60 ± 0.27	0.54 ± 0.25	0.64 ± 0.30	<0.001
Diet Quality Index— International	65.53 ± 9.06	64.77 ± 9.28	63.56 ± 9.65	<0.001
DASH diet score	4.05 ± 1.28	4.05 ± 1.26	3.80 ± 1.22	<0.001
MIND diet score	4.70 ± 0.93	4.63 ± 0.89	4.48 ± 1.00	<0.001
Mediterranean diet score	4.12 ± 1.56	4.15 ± 1.52	3.94 ± 1.57	0.021
DII score	−0.76 ± 1.39	−0.43 ± 1.50	−0.52 ± 1.43	<0.001

Abbreviations: PASE, Physical Activity Scale for the Elderly; SD, standard deviation; DASH, The Dietary Approaches to Stop Hypertension; MIND, The Mediterranean–DASH Intervention for Neurodegenerative Delay; DII, dietary inflammation index. χ^2^ test (categorical variables) and one-way ANOVA (continuous variables) for subgroup differences. *p* < 0.05.

**Table 2:** Associations of sarcopenia and its diagnosis components with MMSE scores among participants in Mr OS and Ms OS study.

	Baseline MMSE (*N* = 3146)	MMSE Change (*N* = 3146)
Sarcopenic Status	Coefficient (95% CI)	Coefficient (95% CI)
Normal	0.00	0.00
Probable sarcopenia	−0.15 (−0.40, 0.11)	−0.51 (−0.76, −0.26) *
Sarcopenia/severe sarcopenia	0.09 (−0.25, 0.42)	−0.52 (−0.85, −0.19) *
Diagnosis components		
Low handgrip strength	−0.25 (−0.61, 0.11)	−0.61 (−0.97, −0.25) *
Handgrip strength (per 1 unit increase)	0.03 (0.01, 0.05) *	0.04 (0.02, 0.06) *
Low physical performance	−0.12 (−0.35, 0.11)	−0.46 (−0.69, −0.23) *
Walking speed (per 1 unit increase)	0.83 (0.30, 1.36) *	0.71 (0.17, 1.24) *
Time to complete 5 stands (per 1 unit increase)	−0.02 (−0.04, 0.01)	−0.04 (−0.07, −0.02) *
Low muscle mass	0.17 (−0.12, 0.46)	−0.03 (−0.32, 0.26)
Muscle mass (per 1 unit increase)	−0.25 (−0.47, −0.02) *	0.12 (−0.11, 0.34)

Abbreviations: MMSE, Mini-Mental State Examination. * *p* < 0.05. The regression model was adjusted for sex, age, dietary energy, body mass index, physical activity, systolic blood pressure, medical history (diabetes, stroke, heart attack, angina, congestive heart failure, or cancer), smoking habit, alcohol drinking, and education level. We also adjusted the baseline MMSE in the model, with MMSE change as the outcome.

**Table 3:** Significant mediating effects of sarcopenia-related components on the association of dietary factors with MMSE scores among all participants in Mr OS and Ms OS study.

	Coefficient (95% CI)	Proportion Mediated (%)	Coefficient (95% CI)	Proportion Mediated (%)
All participants	Baseline MMSE (*N* = 3146)		MMSE change (*N* = 3146)	
**DQI-I**				
Walking speed	0.0011 (0.0003, 0.0023) *	8.04		
Time to complete 5 stands			0.0009 (0.0001, 0.0019) *	9.31
**DII**				
Walking speed	−0.0131 (−0.0248, −0.0030) *	7.43		
Time to complete 5 stands			−0.0100 (−0.0205, −0.0015) *	37.22
**Animal protein**				
Handgrip strength			−0.0324 (−0.0694, −0.0009) *	8.05
**All males**	**Baseline MMSE (*N* = 1564)**			
**Total protein**				
Handgrip strength	−0.0560 (−0.1094, −0.0148) *	13.36		
**DII**				
Time to complete 5 stands			−0.0165 (−0.0328, −0.0030) *	15.05

Abbreviations: DQI-I, Diet Quality Index—International; MMSE, Mini-Mental State Examination; DII, dietary inflammation index. * *p* < 0.05. Indirect associations are presented. Effects are shown with coefficients with 95% CIs.

## Data Availability

The data presented in this study are available on request from the corresponding author due to ethical reasons.

## Supplementary Materials

The following supporting information can be downloaded at: https://www.mdpi.com/article/10.3390/nu17193070/s1. Supplemental Methods: Detailed description of sarcopenia assessment, dietary pattern score calculation, and mediation analysis; Figure S1: Baseline MMSE scores and MMSE change by sarcopenia status; Table S1: Sex-specific levels of energy, protein intake, and adherence to dietary patterns of all participants and participants with sarcopenia or severe sarcopenia; Table S2: Baseline characteristics of participants in Mr. OS and Ms. OS study with or without year-4 MMSE scores; Table S3: Association of dietary protein with MMSE scores among all participants in Mr OS and Ms OS study (full regression results); Table S4: Association of dietary patterns with MMSE scores among all participants in Mr OS and Ms OS study (full regression results); Table S5: Association of dietary protein with MMSE scores among participants with sarcopenia/severe sarcopenia in Mr. OS and Ms. OS study; Table S6: Association of dietary patterns with MMSE scores among participants with sarcopenia/severe sarcopenia in Mr. OS and Ms. OS study; Table S7: Sensitivity analysis excluding BMI and PASE score in the significant diet–cognition models; Table S8: Significant associations of dietary factors with mediators among all participants in Mr. OS and Ms. OS study; Table S9: Quartile cutoff points for protein intake and dietary patterns in different groups.
