# Supplementary material for: Adhering to Healthy Dietary Patterns Prevents Cognitive Decline of Older Adults with Sarcopenia: The Mr. OS and Ms. OS Study
Source: Nutrients. 2025 Sep 26;17(19):3070. doi: 10.3390/nu17193070 (PMC12526030; doi:10.3390/nu17193070)
Supplement: Supplementary file 1 [file nutrients-17-03070-s001.zip › nutrients-3862085-supplementary.pdf]

Supplementary Information

Section 1. Supplemental Methods

Sarcopenia Assessment

Low muscle mass was defined as appendicular skeletal muscle mass (ASM) index (ASM/height^2) <7.0 kg/m^2 in men and <5.4 kg/m^2 in women by dual-energy X-ray absorptiometry (DXA); low muscle strength was defined as grip strength <28 kg for men and <18 kg for women; and low physical performance were 6-m walk <1.0 m/s or 5-time chair stand test ≥12 s for both men and women. All participants were divided into four groups: non-sarcopenia (healthy), possible sarcopenia, sarcopenia, and severe sarcopenia. Possible sarcopenia was defined as either low muscle strength or low physical performance only. Sarcopenia was defined as low ASM, with low muscle strength or low physical performance. Individuals with low ASM, low muscle strength, and low physical performance were diagnosed as severe sarcopenia.

Dietary pattern scores calculation

The Diet Quality Index-International (DQI-I) assessment includes four aspects of the diet with subcomponents: variety, adequacy, moderation and overall balance. The overall score ranges from 0 to 100. Due to insufficient information to calculate the category of empty-calorie foods under the 'moderation' aspect in this study, the scoring range for moderation was adjusted to 0-24 instead of the usual 0-30, and the total score of DQI-I ranged from 0 to 94 instead of 0 to 100, with a high score indicating high quality.

DASH score was calculated based on the method developed by Mellen et al. [1]. The DASH target intakes included nine nutrients (i.e., total fat, saturated fat, protein, fiber, cholesterol, calcium, magnesium, potassium and sodium). One score would be given when achieving each target intake. A score of 0.5 was assigned when achieving a nutrient target between the DASH target and the nutrient content of the control group's diet in the DASH trial. The total score ranged from 0 to 9, with a higher score representing greater adherence.

MIND score was calculated based on 15 food groups associated with the prevention of cognitive impairment: ten beneficial food groups (green leafy vegetables, other vegetables, nuts, berries, beans, whole grains, seafood, poultry, olive oil and wine) and five unhealthy food groups (red meats, butter and stick margarine, cheese, pastries and sweets, and fried/fast food). Each food group was given a score of 0, 0.5, or 1 based on the frequency and proportion of consumption [2]. However, this study lacked sufficient information on the consumption of olive oil and the frequency of consuming fish (not fried), beans, poultry, red meat and products, and fast-fried foods, so the maximum MIND score in our study was 9 instead of 15.

The Mediterranean Diet Score (MDS) was calculated using the methods developed by Trichopoulou et al [3]. One score was given when the consumption of beneficial components (vegetables, legumes, fruits and nuts, cereal, and fish) was at or above the sex-specific median, and the consumption of detrimental components was below the median cutoff. A value of 1 was assigned if daily consumption of ethanol was between 10 and 50 g for men or 5 and 25 g for women.

The dietary inflammatory index (DII) was calculated based on 30 literature-derived food parameters related to chronic inflammation [4]. DII score was calculated as the sum of the standardized scores of 30 food parameters. A higher and positive DII score indicates a more pro-inflammatory diet, while a lower and negative one indicates a more anti-inflammatory diet.

Mediation analysis

Mediation analysis was conducted to explore the potential mediation effect of sarcopenia-related mediators (handgrip strength, walking speed, time to complete 5 stands and muscle mass) and hs-CRP in the association between dietary factors and cognitive outcomes among all participants and participants with sarcopenia/severe sarcopenia. We integrated two regression models, one to regress the cognitive outcomes on the exposure and the mediator, and a linear model to regress the mediator on the exposure, with adjustment for covariates [5 6]. The mediation analysis was performed using the "mediation" package in R. The proportion mediated was estimated in coefficient scale, and 95% CIs were obtained using bootstrapping.

References

1. Mellen PB, Gao SK, Vitolins MZ, et al. Deteriorating dietary habits among adults with hypertension: DASH dietary accordance, NHANES 1988-1994 and 1999-2004. *Arch Intern Med* 2008;168(3):308-14.
2. Morris MC, Tangney CC, Wang Y, et al. MIND diet slows cognitive decline with aging. *Alzheimers Dement* 2015;11(9):1015-22.
3. Trichopoulou A, Costacou T, Bamia C, et al. Adherence to a Mediterranean diet and survival in a Greek population. *The New England journal of medicine* 2003;348(26):2599-608.
4. Shivappa N, Steck SE, Hurley TG, et al. Designing and developing a literature-derived, population-based dietary inflammatory index. *Public Health Nutr* 2014;17(8):1689-96.
5. VanderWeele TJ. Mediation Analysis: A Practitioner's Guide. *Annu Rev Public Health* 2016;37:17-32.
6. Gong JH, Lo K, Liu Q, et al. Dietary Manganese, Plasma Markers of Inflammation, and the Development of Type 2 Diabetes in Postmenopausal Women: Findings From the Women's Health Initiative. *Diabetes Care* 2020;43(6):1344-51.

Section 2. Supplementary Figure and Tables

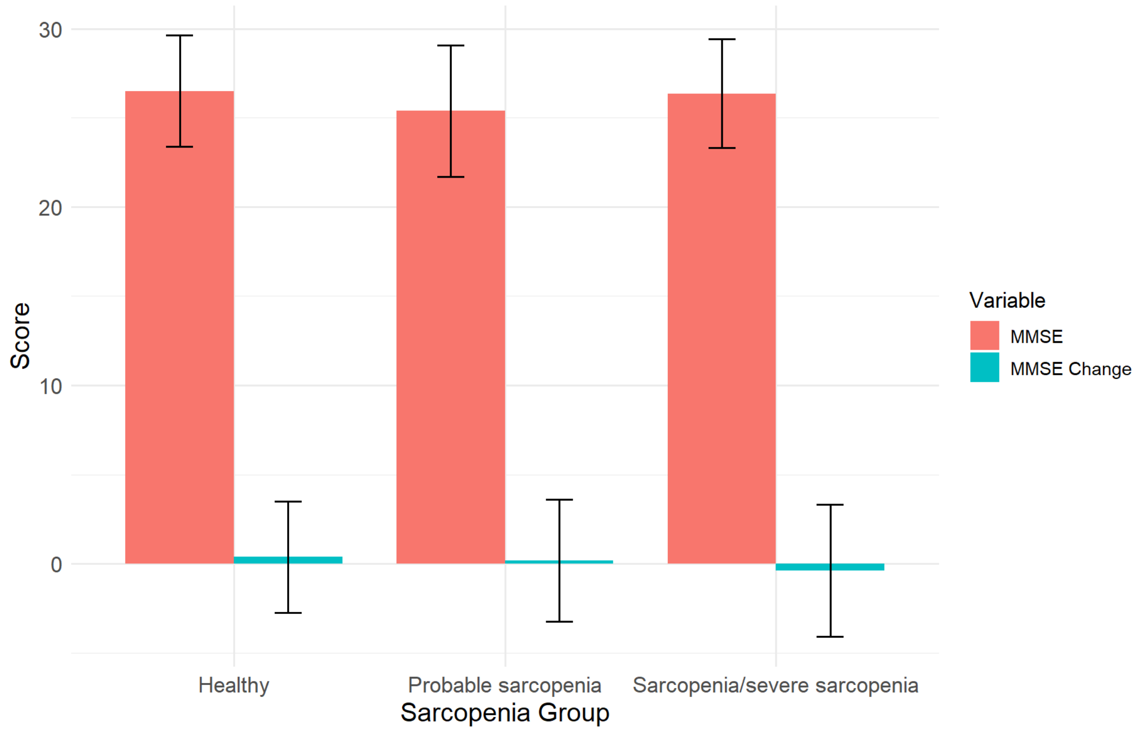

Figure S1 Baseline MMSE Scores and MMSE Change by Sarcopenia Status

Supplementary Table S1 Sex-specific levels of Energy, Protein Intake and Adherence of Dietary Patterns of All Participants and Participants with Sarcopenia or severe sarcopenia

	**All (Males) (N=1,564)**	**All (Females) (N=1,582)**	***P* Value**	**Sarcopenia/Severe Sarcopenia (Males) (N=397)**	**Sarcopenia/Severe Sarcopenia (Females) (N=179)**	***P* Value**
Dietary energy (kcal)	2114.87 ± 584.00	1596.01 ± 458.94	< 0.001*	2062.69 ± 564.39	1595.98 ± 460.02	< 0.001*
Protein (g) per day/kg	1.44 ± 0.59	1.23 ± 0.56	< 0.001*	1.56 ± 0.67	1.41 ± 0.59	0.01*
Animal protein (g) per day/kg	0.84 ± 0.45	0.66 ± 0.42	< 0.001*	0.92 ± 0.52	0.75 ± 0.41	< 0.001*
Plant protein (g) per day/kg	0.59 ± 0.27	0.57 ± 0.26	0.02*	0.63 ± 0.29	0.66 ± 0.32	0.24
Diet Quality Index - International	64.05 ± 9.45	65.51 ± 9.09	< 0.001*	62.74 ± 9.65	65.38 ± 9.42	0.002*
DASH diet score	3.66 ± 1.18	4.35 ± 1.25	< 0.001*	3.59 ± 1.16	4.25 ± 1.25	< 0.001*
MIND diet score	4.56 ± 0.96	4.70 ± 0.89	< 0.001*	4.41 ± 1.03	4.65 ± 0.91	0.007*
Mediterranean diet score	4.11 ± 1.55	4.09 ± 1.53	0.62	3.94 ± 1.57	3.94 ± 1.56	0.96
DII score	-0.88 ± 1.34	-0.22 ± 1.50	< 0.001*	-0.67 ± 1.36	-0.19 ± 1.53	< 0.001*

Presented with Mean ± SD (standard deviation).

Abbreviations: DASH, The Dietary Approaches to Stop Hypertension; MIND, The Mediterranean-DASH Intervention for Neurodegenerative Delay; DII, dietary inflammation index.

Supplementary Table S2 Baseline Characteristics of Participants in Mr OS and Ms OS Study with or without year 4 MMSE scores (excluded in the analysis)

	**No year 4 MMSE score (N=845)**	**With year 4 MMSE score (N=3,146)**	***P* Value**
	Mean (SD)/N (%)		
Sex (Female)	413 (48.9)	1582 (50.3)	0.491
Age (Years)	74.68 ± 5.70	71.90 ± 4.88	<0.001
Post-secondary Education	61 (7.2)	329 (10.5)	0.002
Physical activity (PASE score)	82.38 ± 40.27	93.74 ± 43.40)	<0.001
Smoking habit			0.001
Former smoker	273 (32.3)	916 (29.1)	
Current smoker	78 (9.2)	196 (6.2)	
Drink > 12 alcoholic drinks in the past year	97 (11.5)	425 (13.5)	0.135
Dietary energy (kcal)	1792.99 ± 593.58	1853.96 ± 585.41	0.007
Body mass index (kg/m2)	23.49 ± 3.57	23.74 ± 3.22	0.044
Systolic blood pressure	144.24 ± 20.18	142.20 ± 18.79	0.006
History of diabetes	137 (16.2)	442 (14.0)	0.126
History of stroke	49 (5.8)	124 (3.9)	0.024
History of heart attack	99 (11.7)	293 (9.3)	0.044
History of angina	91 (10.8)	261 (8.3)	0.029
History of congestive heart failure	32 (3.8)	119 (3.8)	1.000
History of cancer	47 (5.6)	129 (4.1)	0.081
Protein (g) per day/kg	1.29 ± 0.62	1.34 ± 0.59	0.052
Animal protein (g) per day/kg	0.74 ± 0.46	0.75 ± 0.44	0.333
Plant protein (g) per day/kg	0.55 ± 0.27	0.58 ± 0.27	0.010
Diet Quality Index - International	62.78 ± 10.40	64.79 ± 9.30	<0.001
DASH diet score	3.87 ± 1.29	4.01 ± 1.26	0.005
MIND diet score	4.40 ± 0.98	4.63 ± 0.93	<0.001
Mediterranean diet score	4.01 ± 1.52	4.10 ± 1.54	0.140
DII score	-0.19 ± 1.56	-0.55 ± 1.46	<0.001

Abbreviations: PASE, Physical Activity Scale for the Elderly; SD, standard deviation; DASH, The Dietary Approaches to Stop Hypertension; MIND, The Mediterranean-DASH Intervention for Neurodegenerative Delay; DII, dietary inflammation index.

$\chi 2$ test (categorical variables) and one-way ANOVA (continuous variables) for subgroup differences. $p < 0.05$.

Supplementary Table S3 Association of Dietary Protein with MMSE Scores Among all Participants in Mr OS and Ms OS Study (full regression results).

	Baseline MMSE			MMSE change		
	All (N=3,146)	Male (N=1,564)	Female (N=1,582)	All (N=3,146)	Male (N=1,564)	Female (N=1,582)
	Coefficient (95% CI)	Coefficient (95% CI)	Coefficient (95% CI)	Coefficient (95% CI)	Coefficient (95% CI)	Coefficient (95% CI)
Total protein intake						
Q1	0.00	0.00	0.00	0.00	0.00	0.00
Q2	0.37 (0.05, 0.68) *	-0.21 (-0.57, 0.14)	0.55 (0.04, 1.06) *	0.36 (0.04, 0.67) *	0.22 (-0.21, 0.66)	0.42 (-0.04, 0.87)
Q3	0.35 (-0.00, 0.71)	-0.17 (-0.57, 0.24)	0.54 (-0.03, 1.10)	0.19 (-0.16, 0.55)	-0.16 (-0.66, 0.33)	0.38 (-0.13, 0.89)
Q4	0.28 (-0.17, 0.74)	-0.42 (-0.92, 0.08)	0.74 (0.01, 1.47) *	0.13 (-0.33, 0.58)	-0.47 (-1.09, 0.15)	0.46 (-0.20, 1.11)
Trend	0.09 (-0.05, 0.24)	-0.12 (-0.28, 0.05)	0.22 (-0.01, 0.46)	0.03 (-0.12, 0.17)	-0.17 (-0.37, 0.02)	0.14 (-0.07, 0.35)
Continuous	0.07 (-0.22, 0.37)	-0.42 (-0.75, -0.09) *†	0.51 (0.02, 1.01) *†	-0.08 (-0.38, 0.22)	-0.23 (-0.63, 0.18)†	-0.03 (-0.48, 0.42)†
Animal protein intake						
Q1	0.00	0.00	0.00	0.00	0.00	0.00
Q2	0.39 (0.09, 0.70) *	0.27 (-0.08, 0.61)	0.42 (-0.06, 0.91)	0.21 (-0.10, 0.51)	0.23 (-0.19, 0.65)	0.15 (-0.28, 0.59)
Q3	0.29 (-0.03, 0.61)	-0.25 (-0.61, 0.12)	0.62 (0.11, 1.14) *	0.14 (-0.18, 0.46)	-0.19 (-0.63, 0.26)	0.08 (-0.38, 0.55)
Q4	0.25 (-0.13, 0.62)	0.03 (-0.39, 0.44)	0.37 (-0.22, 0.96)	-0.10 (-0.48, 0.28)	-0.38 (-0.90, 0.13)	-0.19 (-0.72, 0.34)
Trend	0.07 (-0.05, 0.19)	-0.05 (-0.18, 0.08)	0.14 (-0.04, 0.33)	-0.03 (-0.15, 0.09)	-0.16 (-0.32, 0.01)	-0.06 (-0.22, 0.11)
Continuous	0.09 (-0.22, 0.41)	-0.32 (-0.67, 0.02)†	0.44 (-0.08, 0.96)†	-0.36 (-0.67, -0.05) *	-0.48 (-0.90, -0.06) *†	-0.30 (-0.77, 0.16)†
Plant protein intake						

Q1	0.00	0.00	0.00	0.00	0.00	0.00
Q2	0.00<br>(-0.31, 0.32)	-0.15<br>(-0.50, 0.20)	0.07<br>(-0.44, 0.57)	0.23<br>(-0.08, 0.54)	-0.05<br>(-0.48, 0.38)	0.38<br>(-0.07, 0.84)
Q3	0.10<br>(-0.24, 0.44)	-0.35<br>(-0.73, 0.03)	0.53<br>(-0.02, 1.08)	0.33<br>(-0.01, 0.67)	0.13<br>(-0.35, 0.60)	0.50<br>(0.01, 1.00) *
Q4	0.02<br>(-0.39, 0.42)	-0.48<br>(-0.93, -0.03) *	0.36<br>(-0.32, 1.03)	0.45<br>(0.05, 0.86) *	0.04<br>(-0.51, 0.58)	0.78<br>(0.18, 1.39) *
Trend	0.02<br>(-0.11, 0.15)	-0.16<br>(-0.31, -0.02) *	0.17<br>(-0.05, 0.38)	0.15<br>(0.02, 0.28) *	0.03<br>(-0.15, 0.21)	0.25<br>(0.05, 0.44) *
Continuous	-0.05<br>(-0.60, 0.51)	-0.37<br>(-0.98, 0.23)†	0.38<br>(-0.56, 1.32)†	0.79<br>(0.24, 1.35) *	0.66<br>(-0.08, 1.40)†	0.88<br>(0.04, 1.73) *†

Abbreviations: OR, odds ratio; MMSE, Mini-Mental State Examination. *p<0.05.

The regression model was adjusted for sex, age, dietary energy, body mass index, physical activity, systolic blood pressure, medical history (diabetes, stroke, heart attack, angina, congestive heart failure or cancer), smoking habit, alcohol drinking, education level. Also adjusted baseline MMSE in model with MMSE change as outcome. †Sex×exposure interaction (tested on the continuous term) is significant at P<0.05. Sex-stratified estimates are exploratory.

Supplementary Table S4 Association of Dietary Patterns with MMSE Scores Among all Participants in Mr OS and Ms OS Study (full regression results).

	Baseline MMSE			MMSE change		
	All (N=3,146)	Male (N=1,564)	Female (N=1,582)	All (N=3,146)	Male (N=1,564)	Female (N=1,582)
	Coefficient (95% CI)	Coefficient (95% CI)	Coefficient (95% CI)	Coefficient (95% CI)	Coefficient (95% CI)	Coefficient (95% CI)
DQI-I						
Q1	0.00	0.00	0.00	0.00	0.00	0.00
Q2	0.33 (0.04, 0.62) *	0.21 (-0.11, 0.54)	0.37 (-0.09, 0.84)	0.10 (-0.19, 0.39)	0.09 (-0.30, 0.49)	0.23 (-0.19, 0.64)
Q3	0.21 (-0.09, 0.52)	0.30 (-0.04, 0.63)	0.06 (-0.44, 0.56)	0.33 (0.02, 0.63) *	0.18 (-0.23, 0.59)	0.39 (-0.06, 0.83)
Q4	0.21 (-0.10, 0.51)	-0.02 (-0.37, 0.32)	0.28 (-0.23, 0.79)	0.34 (0.03, 0.64) *	0.21 (-0.21, 0.64)	0.35 (-0.10, 0.81)
Trend	0.05 (-0.04, 0.15)	0.01 (-0.10, 0.12)	0.05 (-0.11, 0.22)	0.12 (0.03, 0.22) *	0.07 (-0.06, 0.21)	0.12 (-0.02, 0.27)
Continuous	0.01 (0.00, 0.03) *	0.01 (-0.01, 0.02)	0.02 (-0.00, 0.04)	0.02 (0.00, 0.03) *	0.01 (-0.00, 0.03)	0.02 (0.00, 0.04) *
DASH						
Q1	0.00	0.00	0.00	0.00	0.00	0.00
Q2	0.05 (-0.22, 0.33)	-0.03 (-0.39, 0.32)	-0.26 (-0.69, 0.17)	0.03 (-0.25, 0.30)	0.46 (0.03, 0.89) *	0.16 (-0.22, 0.55)
Q3	0.18 (-0.12, 0.47)	0.03 (-0.26, 0.32)	0.20 (-0.35, 0.74)	0.22 (-0.08, 0.51)	-0.02 (-0.37, 0.34)	0.60 (0.12, 1.09) *
Q4	0.33 (-0.01, 0.67)	0.04 (-0.30, 0.39)	0.19 (-0.29, 0.67)	0.29 (-0.05, 0.63)	-0.02 (-0.44, 0.40)	0.46 (0.03, 0.89) *
Trend	0.11 (0.00, 0.21) *	0.02 (-0.09, 0.12)	0.09 (-0.06, 0.25)	0.11 (0.00, 0.21) *	-0.02 (-0.15, 0.11)	0.18 (0.04, 0.32) *
Continuous	0.06 (-0.02, 0.15)	0.01 (-0.09, 0.11)	0.10 (-0.04, 0.24)	0.07 (-0.02, 0.16)	-0.01 (-0.13, 0.12)	0.14 (0.02, 0.26) *
MIND						
Q1	0.00	0.00	0.00	0.00	0.00	0.00
Q2	0.02 (-0.29, 0.32)	-0.15 (-0.49, 0.19)	0.31 (-0.11, 0.72)	0.32 (0.02, 0.63) *	0.22 (-0.20, 0.63)	0.39 (0.02, 0.76) *
Q3	0.22 (-0.04, 0.48)	-0.08 (-0.42, 0.25)	0.48 (-0.07, 1.02)	0.31 (0.05, 0.58) *	0.44 (0.02, 0.85) *	0.09 (-0.40, 0.58)
Q4	0.18 (-0.18, 0.55)	0.01 (-0.31, 0.34)	0.50 (-0.09, 1.10)	0.25 (-0.12, 0.61)	0.35 (-0.04, 0.75)	0.06 (-0.47, 0.59)

Trend	0.09<br>(-0.02, 0.19)	0.00<br>(-0.10, 0.11)	0.18<br>(0.00, 0.36) *	0.11<br>(0.00, 0.21) *	0.13<br>(0.01, 0.26) *	0.01<br>(-0.15, 0.17)
Continuous	0.09<br>(-0.02, 0.21)	0.00<br>(-0.13, 0.13)†	0.21<br>(0.00, 0.41) *†	0.12<br>(-0.00, 0.24)	0.18<br>(0.02, 0.34) *†	0.05<br>(-0.13, 0.23)†
MDS						
Q1	0.00	0.00	0.00	0.00	0.00	0.00
Q2	0.28<br>(0.01, 0.56) *	0.04<br>(-0.27, 0.35)	0.54<br>(0.10, 0.99)	-0.04<br>(-0.32, 0.23)	0.06<br>(-0.32, 0.44)	-0.16<br>(-0.56, 0.24)
Q3	0.05<br>(-0.25, 0.35)	0.10<br>(-0.24, 0.43)	0.07<br>(-0.42, 0.56)	0.08<br>(-0.22, 0.38)	0.37<br>(-0.04, 0.78)	-0.19<br>(-0.63, 0.25)
Q4	-0.06<br>(-0.37, 0.25)	-0.05<br>(-0.41, 0.30)	-0.10<br>(-0.61, 0.40)	0.13<br>(-0.18, 0.45)	0.44<br>(0.01, 0.87) *	-0.20<br>(-0.65, 0.26)
Trend	-0.02<br>(-0.12, 0.07)	-0.00<br>(-0.11, 0.11)	-0.05<br>(-0.20, 0.11)	0.05<br>(-0.05, 0.15)	0.16<br>(0.03, 0.30) *	-0.07<br>(-0.21, 0.07)
Continuous	-0.03<br>(-0.10, 0.04)	0.01<br>(-0.07, 0.09)	-0.07<br>(-0.19, 0.05)	0.03<br>(-0.04, 0.10)	0.11<br>(0.01, 0.21) *	-0.06<br>(-0.16, 0.05)
DII						
Q1	0.00	0.00	0.00	0.00	0.00	0.00
Q2	0.07<br>(-0.23, 0.38)	0.12<br>(-0.22, 0.47)	0.14<br>(-0.36, 0.64)	-0.30<br>(-0.60, 0.01)	-0.34<br>(-0.76, 0.08)	-0.24<br>(-0.69, 0.21)
Q3	-0.15<br>(-0.47, 0.17)	0.18<br>(-0.18, 0.54)	-0.46<br>(-0.99, 0.06)	-0.16<br>(-0.48, 0.17)	-0.33<br>(-0.77, 0.12)	-0.13<br>(-0.60, 0.34)
Q4	-0.54<br>(-0.89, -0.19) *	-0.07<br>(-0.46, 0.32)	-0.93<br>(-1.48, -0.37) *	-0.35<br>(-0.70, -0.00) *	-0.27<br>(-0.74, 0.21)	-0.24<br>(-0.74, 0.26)
Trend	-0.19<br>(-0.30, -0.08) *	-0.02<br>(-0.14, 0.10)	-0.35<br>(-0.52, -0.17) *	-0.09<br>(-0.20, 0.02)	-0.07<br>(-0.23, 0.08)	-0.06<br>(-0.22, 0.10)
Continuous	-0.18<br>(-0.26, -0.09) *	-0.04<br>(-0.14, 0.07)†	-0.28<br>(-0.41, -0.14) *†	-0.10<br>(-0.19, -0.02) *	-0.13<br>(-0.26, -0.00) *†	-0.06<br>(-0.19, 0.06)†

Abbreviations: MMSE, Mini-Mental State Examination; DQI-I, Diet Quality Index - International; DASH, Dietary Approaches to Stop Hypertension; MIND, Mediterranean-DASH Diet Intervention for Neurodegenerative Delay; MDS, Mediterranean Diet Score; DII, dietary inflammation index; OR, odds ratio. *p<0.05. The regression model was adjusted for sex, age, dietary energy, body mass index, physical activity, systolic blood pressure, medical history (diabetes, stroke, heart attack, angina, congestive heart failure or cancer), smoking habit, alcohol drinking, education level. Also adjusted baseline MMSE in model with MMSE change as outcome.

Supplementary Table S5 Association of Dietary Protein with MMSE Scores Among Participants with Sarcopenia/Severe Sarcopenia in Mr OS and Ms OS Study

	**Baseline MMSE**			**MMSE change**		
	All (N=576)	Male (N=397)	Female (N=179)	All (N=576)	Male (N=397)	Female (N=179)
	Coefficient (95% CI)	Coefficient (95% CI)	Coefficient (95% CI)	Coefficient (95% CI)	Coefficient (95% CI)	Coefficient (95% CI)
Total protein intake						
**Q1**	0.00	0.00	0.00	0.00	0.00	0.00
**Q2**	-0.14 (-0.82, 0.54)	-0.42 (-1.17, 0.34)	0.09 (-1.35, 1.52)	0.43 (-0.39, 1.26)	0.19 (-0.79, 1.16)	0.69 (-0.88, 2.25)
**Q3**	-0.22 (-0.97, 0.53)	-0.34 (-1.17, 0.49)	-0.43 (-1.99, 1.14)	-0.35 (-1.25, 0.56)	-0.92 (-1.99, 0.16)	0.64 (-1.07, 2.35)
**Q4**	-0.36 (-1.33, 0.62)	-0.58 (-1.66, 0.50)	-0.49 (-2.49, 1.52)	-0.23 (-1.41, 0.96)	-0.67 (-2.07, 0.73)	0.99 (-1.21, 3.18)
**Trend**	-0.11 (-0.42, 0.19)	-0.17 (-0.51, 0.18)	-0.19 (-0.82, 0.43)	-0.15 (-0.52, 0.23)	-0.34 (-0.79, 0.10)	0.30 (-0.38, 0.99)
**Continuous**	-0.36 (-0.95, 0.22)	-0.34 (-0.95, 0.27)	-0.54 (-2.03, 0.95)	-0.07 (-0.78, 0.64)	-0.31 (-1.11, 0.48)	0.31 (-1.32, 1.95)
Animal protein intake						
**Q1**	0.00	0.00	0.00	0.00	0.00	0.00
**Q2**	0.36 (-0.30, 1.03)	-0.28 (-1.00, 0.45)	0.22 (-1.25, 1.68)	0.36 (-0.44, 1.17)	0.49 (-0.45, 1.43)	-0.54 (-2.14, 1.05)
**Q3**	-0.09 (-0.79, 0.60)	-0.30 (-1.08, 0.47)	0.48 (-1.00, 1.97)	-0.09 (-0.94, 0.75)	-0.46 (-1.46, 0.55)	-0.73 (-2.35, 0.89)
**Q4**	0.41 (-0.40, 1.23)	0.10 (-0.78, 0.98)	0.27 (-1.42, 1.96)	-0.25 (-1.24, 0.74)	-0.52 (-1.67, 0.62)	0.52 (-1.32, 2.36)
**Trend**	0.07 (-0.19, 0.33)	0.02 (-0.27, 0.30)	0.12 (-0.41, 0.65)	-0.11 (-0.43, 0.20)	-0.25 (-0.61, 0.12)	0.10 (-0.48, 0.68)
**Continuous**	-0.09 (-0.69, 0.51)	-0.24 (-0.86, 0.38)	0.44 (-1.11, 1.99)	-0.23 (-0.95, 0.50)	-0.49 (-1.30, 0.32)	0.26 (-1.44, 1.96)
Plant protein intake						
**Q1**	0.00	0.00	0.00	0.00	0.00	0.00
**Q2**	-0.24 (-0.92, 0.44)	-0.24 (-1.00, 0.51)	-0.52 (-1.97, 0.93)	-0.27 (-1.09, 0.56)	-0.04 (-1.02, 0.95)	-0.35 (-1.93, 1.24)
**Q3**	-0.10 (-0.84, 0.64)	-0.24 (-1.06, 0.58)	-0.77 (-2.37, 0.84)	-0.06 (-0.96, 0.84)	-0.42 (-1.49, 0.64)	1.16 (-0.60, 2.91)
**Q4**	-0.29 (-1.18, 0.59)	-0.48 (-1.44, 0.48)	-1.77 (-3.74, 0.21)	-0.11 (-1.19, 0.96)	0.01 (-1.24, 1.25)	-0.26 (-2.43, 1.92)
**Trend**	-0.07 (-0.35, 0.21)	-0.14 (-0.45, 0.16)	-0.54 (-1.17, 0.10)	-0.01 (-0.36, 0.33)	-0.04 (-0.44, 0.35)	0.13 (-0.58, 0.83)
**Continuous**	-0.94 (-2.02, 0.13)	-0.45 (-1.66, 0.76)	-2.25 (-4.53, 0.02)	0.48 (-0.83, 1.79)	0.59 (-0.99, 2.16)	0.18 (-2.37, 2.74)

Abbreviations: OR, odds ratio; MMSE, Mini-Mental State Examination. *p<0.05.
The regression model was adjusted for sex, age, dietary energy, body mass index, physical activity, systolic blood pressure, medical history (diabetes, stroke, heart attack, angina, congestive heart failure or cancer), smoking habit, alcohol drinking, education level. Also adjusted baseline MMSE in model with MMSE change as outcome.

Supplementary Table S6 Association of Dietary Patterns with MMSE Scores Among Participants with Sarcopenia/Severe Sarcopenia in Mr OS and Ms OS Study

	**Baseline MMSE**			**MMSE change**		
	All (N=576)	Male (N=397)	Female (N=179)	All (N=576)	Male (N=397)	Female (N=179)
	Coefficient (95% CI)	Coefficient (95% CI)	Coefficient (95% CI)	Coefficient (95% CI)	Coefficient (95% CI)	Coefficient (95% CI)
DQI-I						
**Q1**	0.00	0.00	0.00	0.00	0.00	0.00
**Q2**	0.69 (0.04, 1.34) *	0.83 (0.11, 1.55) *	0.93 (-0.43, 2.29)	-0.25 (-1.06, 0.55)	-0.19 (-1.15, 0.76)	0.99 (-0.52, 2.50)
**Q3**	0.86 (0.21, 1.51) *	0.84 (0.13, 1.55) *	-0.18 (-1.59, 1.24)	-0.29 (-1.10, 0.51)	-0.07 (-1.01, 0.87)	0.46 (-1.11, 2.02)
**Q4**	-0.17 (-0.88, 0.53)	0.25 (-0.50, 0.99)	-0.81 (-2.38, 0.76)	0.12 (-0.75, 0.98)	-0.17 (-1.16, 0.81)	1.20 (-0.54, 2.94)
**Trend**	-0.02 (-0.24, 0.20)	0.08 (-0.15, 0.32)	-0.32 (-0.81, 0.18)	0.02 (-0.25, 0.29)	-0.04 (-0.35, 0.27)	0.30 (-0.24, 0.85)
**Continuous**	0.00 (-0.02, 0.03)	0.02 (-0.01, 0.04)	-0.03 (-0.09, 0.03)	0.01 (-0.02, 0.04)	0.00 (-0.04, 0.04)	0.03 (-0.03, 0.09)
DASH						
**Q1**	0.00	0.00	0.00	0.00	0.00	0.00
**Q2**	0.73 (0.16, 1.30) *	-0.25 (-0.93, 0.44)	-0.29 (-1.77, 1.18)	0.23 (-0.47, 0.93)	0.45 (-0.45, 1.34)	0.87 (-0.74, 2.49)
**Q3**	0.16 (-0.61, 0.93)	0.33 (-0.36, 1.01)	-0.98 (-2.25, 0.29)	0.55 (-0.39, 1.49)	0.24 (-0.66, 1.13)	1.09 (-0.31, 2.50)
**Q4**	0.08 (-0.59, 0.74)	0.32 (-0.52, 1.17)	-1.43 (-2.89, 0.02)	0.58 (-0.23, 1.39)	-0.03 (-1.13, 1.08)	1.30 (-0.32, 2.91)
**Trend**	-0.01 (-0.22, 0.21)	0.16 (-0.09, 0.42)	-0.49 (-0.93, -0.05) *	0.20 (-0.05, 0.46)	-0.00 (-0.33, 0.33)	0.45 (-0.04, 0.94)
**Continuous**	0.06 (-0.14, 0.26)	0.20 (-0.02, 0.42)†	-0.25 (-0.66, 0.15)†	0.17 (-0.07, 0.41)	-0.00 (-0.29, 0.29)†	0.40 (-0.04, 0.85)†
MIND						
**Q1**	0.00	0.00	0.00	0.00	0.00	0.00
**Q2**	-0.38 (-0.99, 0.23)	-0.60 (-1.25, 0.04)	-0.75 (-2.22, 0.71)	0.91 (0.18, 1.65) *	0.76 (-0.08, 1.61)	0.97 (-0.63, 2.58)
**Q3**	-0.07 (-0.82, 0.68)	0.01 (-0.82, 0.84)	-0.43 (-1.72, 0.86)	0.99 (0.08, 1.89) *	0.57 (-0.51, 1.65)	1.03 (-0.39, 2.44)
**Q4**	-0.25 (-0.94, 0.44)	-0.53 (-1.29, 0.22)	0.08 (-1.63, 1.79)	1.19 (0.36, 2.03) *	1.16 (0.18, 2.14) *	0.30 (-1.56, 2.16)
**Trend**	-0.05 (-0.27, 0.17)	-0.12 (-0.36, 0.13)	-0.04 (-0.55, 0.46)	0.36 (0.09, 0.63) *	0.34 (0.03, 0.66) *	0.24 (-0.31, 0.79)
**Continuous**	-0.12 (-0.37, 0.13)	-0.16 (-0.43, 0.11)	-0.04 (-0.63, 0.55)	0.43 (0.12, 0.73) *	0.38 (0.04, 0.73) *	0.42 (-0.22, 1.06)
MDS						
**Q1**	0.00	0.00	0.00	0.00	0.00	0.00
**Q2**	0.40 (-0.18, 0.98)	0.11 (-0.53, 0.75)	0.95 (-0.30, 2.20)	0.58 (-0.13, 1.28)	0.35 (-0.47, 1.18)	1.00 (-0.41, 2.40)
**Q3**	-0.04 (-0.72, 0.63)	0.34 (-0.39, 1.07)	-0.82 (-2.34, 0.69)	1.45 (0.64, 2.27) *	1.42 (0.48, 2.36) *	1.50 (-0.20, 3.19)
**Q4**	-0.53 (-1.25, 0.18)	-0.25 (-1.05, 0.55)	-1.24 (-2.70, 0.23)	0.74 (-0.12, 1.60)	0.95 (-0.09, 1.98)	-0.07 (-1.72, 1.57)
**Trend**	-0.16 (-0.38, 0.06)	-0.02 (-0.26, 0.23)	-0.47 (-0.94, 0.00)	0.36 (0.09, 0.63) *	0.43 (0.11, 0.75) *	0.10 (-0.42, 0.63)
**Continuous**	-0.13 (-0.28, 0.03)	-0.02 (-0.19, 0.15)†	-0.39 (-0.73, -0.05) *†	0.24 (0.05, 0.42) *	0.27 (0.05, 0.49) *†	0.03 (-0.35, 0.42)†
DII						
**Q1**	0.00	0.00	0.00		0.00	0.00
**Q2**	-0.30 (-0.99, 0.38)	0.07 (-0.67, 0.82)	-0.78 (-2.29, 0.73)	-0.48 (-1.31, 0.35)	-0.78 (-1.74, 0.19)	0.15 (-1.50, 1.81)
**Q3**	-0.32 (-1.05, 0.40)	-0.10 (-0.89, 0.70)	-0.42 (-1.99, 1.15)	-0.53 (-1.40, 0.35)	-1.00 (-2.03, 0.03)	-0.11 (-1.83, 1.61)

**Q4**	-0.19 (-0.98, 0.60)	-0.04 (-0.89, 0.81)	-0.44 (-2.16, 1.29)	-0.71 (-1.66, 0.25)	-0.83 (-1.93, 0.27)	-0.54 (-2.43, 1.35)
**Trend**	-0.05 (-0.30, 0.20)	-0.03 (-0.30, 0.24)	-0.06 (-0.60, 0.47)	-0.21 (-0.51, 0.09)	-0.26 (-0.61, 0.09)	-0.20 (-0.79, 0.39)
**Continuous**	-0.08 (-0.28, 0.11)	-0.10 (-0.32, 0.12)	0.04 (-0.38, 0.45)	-0.26 (-0.50, -0.02) *	-0.30 (-0.59, -0.01) *	-0.12 (-0.57, 0.33)

Abbreviations: MMSE, Mini-Mental State Examination; DQI-I, Diet Quality Index - International; DASH, Dietary Approaches to Stop Hypertension; MIND, Mediterranean-DASH Diet Intervention for Neurodegenerative Delay; MDS, Mediterranean Diet Score; DII, dietary inflammation index; OR, odds ratio. *p<0.05. The regression model was adjusted for sex, age, dietary energy, body mass index, physical activity, systolic blood pressure, medical history (diabetes, stroke, heart attack, angina, congestive heart failure or cancer), smoking habit, alcohol drinking, education level. Also adjusted baseline MMSE in model with MMSE change as outcome. [†]Sex×exposure interaction (tested on the continuous term) is significant at P<0.05. Sex-stratified estimates are exploratory.

Supplementary Table S7 Sensitivity analysis-exclude BMI and PASE score in the significant diet-cognition models

	Baseline MMSE			MMSE change		
	All	Male	Female	All	Male	Female
	Coefficient (95% CI)	Coefficient (95% CI)	Coefficient (95% CI)	Coefficient (95% CI)	Coefficient (95% CI)	Coefficient (95% CI)
**All participants (N=3,146)**						
Plant protein intake						
**Q1**				0.00	0.00	0.00
**Q2**				0.20 (-0.11, 0.50)	-0.08 (-0.50, 0.35)	0.35 (-0.10, 0.79)
**Q3**				0.29 (-0.04, 0.61)	0.07 (-0.39, 0.52)	0.46 (-0.01, 0.93)
**Q4**				0.38 (0.01, 0.76) *	-0.05 (-0.56, 0.47)	0.69 (0.13, 1.25) *
**Trend**				0.12 (0.00, 0.25) *	0.00 (-0.16, 0.17)	0.22 (0.04, 0.39) *
**Continuous**				0.69 (0.17, 1.21) *	0.47 (-0.22, 1.16)	0.81 (0.03, 1.60) *
DQI-I						
**Q1**				0.00	0.00	0.00
**Q2**				0.09 (-0.20, 0.38)	0.09 (-0.30, 0.49)	0.21 (-0.21, 0.62)
**Q3**				0.32 (0.02, 0.62) *	0.18 (-0.23, 0.59)	0.35 (-0.09, 0.80)
**Q4**				0.32 (0.02, 0.63) *	0.21 (-0.21, 0.64)	0.33 (-0.13, 0.78)
**Trend**				0.12 (0.02, 0.22) *	0.07 (-0.06, 0.21)	0.12 (-0.03, 0.26)
**Continuous**				0.02 (0.00, 0.03) *	0.01 (-0.00, 0.03)	0.02 (0.00, 0.04) *
DASH						
**Q1**				0.00	0.00	0.00
**Q2**				0.02 (-0.26, 0.29)	0.46 (0.03, 0.89) *	0.15 (-0.23, 0.54)
**Q3**				0.21 (-0.08, 0.51)	-0.02 (-0.37, 0.34)	0.58 (0.09, 1.07) *
**Q4**				0.28 (-0.06, 0.62)	-0.02 (-0.44, 0.40)	0.42 (-0.01, 0.85)

**Trend**				0.10 (-0.00, 0.21)	-0.02 (-0.15, 0.11)	0.13 (0.00, 0.25) *
**Continuous**				0.07 (-0.02, 0.15)	-0.01 (-0.13, 0.11)	0.16 (0.03, 0.30) *
MIND						
**Q1**	0.00	0.00	0.00	0.00	0.00	0.00
**Q2**	0.02 (-0.28, 0.33)	-0.13 (-0.47, 0.21)	0.30 (-0.11, 0.72)	0.31 (0.01, 0.62) *	0.23 (-0.19, 0.64)	0.34 (-0.03, 0.71)
**Q3**	0.22 (-0.04, 0.48)	-0.08 (-0.41, 0.26)	0.49 (-0.06, 1.03)	0.30 (0.04, 0.56) *	0.44 (0.03, 0.85) *	0.04 (-0.44, 0.53)
**Q4**	0.19 (-0.17, 0.56)	0.03 (-0.29, 0.36)	0.51 (-0.08, 1.10)	0.22 (-0.14, 0.59)	0.36 (-0.04, 0.75)	-0.00 (-0.53, 0.52)
**Trend**	0.09 (-0.02, 0.19)	0.01 (-0.09, 0.11)	0.19 (0.00, 0.37) *	0.10 (-0.00, 0.20)	0.13 (0.01, 0.26) *	-0.01 (-0.17, 0.15)
**Continuous**	0.10 (-0.02, 0.22)	0.01 (-0.12, 0.14)	0.21 (0.01, 0.41) *	0.11 (-0.01, 0.23)	0.19 (0.03, 0.35) *	0.03 (-0.15, 0.20)
MDS						
**Q1**				0.00	0.00	0.00
**Q2**				-0.04 (-0.32, 0.23)	0.06 (-0.32, 0.44)	-0.15 (-0.55, 0.26)
**Q3**				0.07 (-0.23, 0.37)	0.37 (-0.04, 0.78)	-0.21 (-0.65, 0.23)
**Q4**				0.12 (-0.19, 0.43)	0.45 (0.02, 0.88) *	-0.22 (-0.67, 0.23)
**Trend**				0.04 (-0.05, 0.14)	0.16 (0.03, 0.30) *	-0.08 (-0.22, 0.06)
**Continuous**				0.02 (-0.05, 0.10)	0.11 (0.02, 0.21) *	-0.07 (-0.17, 0.04)
DII						
**Q1**	0.00	0.00	0.00			
**Q2**	0.07 (-0.24, 0.37)	0.11 (-0.24, 0.45)	0.13 (-0.37, 0.63)			
**Q3**	-0.15 (-0.48, 0.17)	0.15 (-0.21, 0.51)	-0.46 (-0.99, 0.06)			
**Q4**	-0.55 (-0.90, -0.20) *	-0.10 (-0.49, 0.29)	-0.92 (-1.47, -0.36) *			
**Trend**	-0.19 (-0.30, -0.08) *	-0.03 (-0.15, 0.10)	-0.34 (-0.52, -0.16) *			
**Continuous**	-0.18 (-0.26, -0.09) *	-0.04 (-0.15, 0.06)	-0.27 (-0.41, -0.14) *			
**Participants with sarcopenia (N=576)**						
MIND						

**Q1**	0.00	0.00	0.00
**Q2**	0.91 (0.17, 1.64) *	0.76 (-0.08, 1.60)	1.00 (-0.57, 2.58)
**Q3**	1.00 (0.10, 1.90) *	0.56 (-0.51, 1.64)	1.09 (-0.30, 2.47)
**Q4**	1.21 (0.38, 2.04) *	1.17 (0.20, 2.14) *	0.27 (-1.58, 2.12)
**Trend**	0.37 (0.10, 0.63) *	0.35 (0.03, 0.66) *	0.25 (-0.30, 0.80)
**Continuous**	0.43 (0.13, 0.74) *	0.39 (0.04, 0.74) *	0.42 (-0.22, 1.06)
MDS			
**Q1**	0.00	0.00	0.00
**Q2**	0.57 (-0.13, 1.27)	0.36 (-0.47, 1.18)	0.92 (-0.48, 2.31)
**Q3**	1.46 (0.65, 2.28) *	1.43 (0.49, 2.37) *	1.40 (-0.26, 3.06)
**Q4**	0.75 (-0.10, 1.61)	0.96 (-0.06, 1.98)	-0.12 (-1.75, 1.51)
**Trend**	0.37 (0.10, 0.63) *	0.43 (0.11, 0.75) *	0.09 (-0.43, 0.61)
**Continuous**	0.24 (0.05, 0.43) *	0.27 (0.06, 0.49) *	0.03 (-0.35, 0.41)

Supplementary Table S8 Significant Associations of Dietary Factors with Mediators Among All Participants in Mr OS and Ms OS Study

	Handgrip strength	Walking speed	Time to complete 5 stands
	Coefficient (95% CI)	Coefficient (95% CI)	Coefficient (95% CI)
**All Participants**			
Animal protein	-1.28 (-1.82, -0.74) *		
DQI-I		0.00 (0.00, 0.00) *	-0.03 (-0.04, -0.01) *
DII		-0.02 (-0.02, -0.01) *	0.29 (0.17, 0.41) *
**All-Males**			
Total protein	-1.74 (-2.59, -0.89) *		
Animal protein	-1.62 (-2.50 -0.75) *		
DII			0.23 (0.07, 0.39) *
**All-Females**			
MIND		0.01 (0.00, 0.02) *	
DII		-0.02 (-0.02, -0.01) *	

Only dietary factors and mediators with significant effect on outcomes (MMSE/MMSE change) were presented.
Abbreviations: DQI-I, Diet Quality Index - International; DASH, Dietary Approaches to Stop Hypertension; MIND, Mediterranean-DASH Diet Intervention for Neurodegenerative Delay; DII, dietary inflammation index. *p<0.05. The linear regression model was adjusted for sex, age, dietary energy, body mass index, physical activity, systolic blood pressure, medical history (diabetes, stroke, heart attack, angina, congestive heart failure or cancer), smoking habit, alcohol drinking, education level. Also adjusted baseline MMSE in model with MMSE change as outcome.

Supplementary Table S9 Quartiles Cutoff points for protein intake and dietary patterns in different group

	Q1	Q2	Q3	Q4
**All participants**				
Total protein	≤ 0.91	> 0.91 to ≤1.23	> 1.23 to ≤1.62	> 1.62
Animal protein	≤ 0.45	> 0.45 to ≤0.66	> 0.66 to ≤0.95	> 0.95
Plant protein	≤ 0.40	> 0.40 to ≤0.52	> 0.52 to ≤0.69	> 0.69
DQI-I (out of 94)	≤ 59	> 59 to ≤66	> 66 to ≤71	> 71
DASH (out of 9)	≤ 2.50	≥ 3.00 to ≤3.50	≥ 4.00 to ≤4.50	≥ 5.00
MIND (out of 9)	≤ 3.50	≥ 4.00 to ≤4.00	≥ 4.50 to ≤4.50	≥ 5.00
MDS (out of 9)	≤ 2.50	≥ 3.00 to ≤3.00	≥ 4.00 to ≤4.50	≥ 5.00
DII	≤ -1.57	> -1.57 to ≤ -0.62	> -0.62 to ≤ 0.35	> 0.35
**All males**				
Total protein	≤ 1.01	> 1.01 to ≤1.34	> 1.34 to ≤1.74	> 1.74
Animal protein	≤ 0.52	> 0.52 to ≤0.75	> 0.75 to ≤1.05	> 1.05
Plant protein	≤ 0.41	> 0.41 to ≤0.53	> 0.53 to ≤0.70	> 0.70
DQI-I (out of 94)	≤ 58	> 58 to ≤65	> 65 to ≤71	> 71
DASH (out of 9)	≤ 3.00	> 3.00 to ≤3.50	> 3.50 to ≤4.50	> 4.50
MIND (out of 9)	≤ 4.00	> 4.00 to ≤4.50	> 4.50 to ≤5.00	> 5.00
MDS (out of 9)	≤ 3.00	> 3.00 to ≤4.00	> 4.00 to ≤5.00	> 5.00
DII	≤ -1.76	> -1.76 to ≤-0.83	> -0.83 to ≤0.05	> 0.05
**All females**				
Total protein	≤ 0.85	> 0.85 to ≤1.13	> 1.13 to ≤1.48	> 1.48
Animal protein	≤ 0.40	> 0.40 to ≤0.58	> 0.58 to ≤0.83	> 0.83
Plant protein	≤ 0.39	> 0.39 to ≤0.51	> 0.51 to ≤0.68	> 0.68
DQI-I (out of 94)	≤ 60	> 60 to ≤67	> 67 to ≤72	> 72
DASH (out of 9)	≤ 3.50	> 3.50 to ≤4.50	> 4.50 to ≤5.00	> 5.00
MIND (out of 9)	≤ 4.00	> 4.00 to ≤5.00	> 5.00 to ≤5.50	> 5.50
MDS (out of 9)	≤ 3	> 3 to ≤4	> 4 to ≤5	> 5
DII	≤ -1.28	> -1.28 to ≤-0.31	> -0.31 to ≤0.77	> 0.77
**Participants with Moderate/severe sarcopenia**				
Total protein	≤ 1.04	> 1.04 to ≤1.39	> 1.39 to ≤1.84	> 1.84
Animal protein	≤ 0.52	> 0.52 to ≤0.77	> 0.77 to ≤1.08	> 1.08
Plant protein	≤ 0.43	> 0.43 to ≤0.56	> 0.56 to ≤0.77	> 0.77
DQI-I (out of 94)	≤ 57	> 57 to ≤64	> 64 to ≤71	> 71
DASH (out of 9)	≤ 3	> 3 to ≤4	> 4 to ≤4.5	> 4.5
MIND (out of 9)	≤ 3.5	> 3.5 to ≤4.5	> 4.5 to ≤5	> 5
MDS (out of 9)	≤ 3	> 3 to ≤4	> 4 to ≤5	> 5

DII	≤ -1.56	> -1.56 to ≤-0.51	> -0.51 to ≤0.35	> 0.35
**Male participants with Moderate/severe sarcopenia**				
Total protein	≤ 1.07	> 1.07 to ≤1.45	> 1.45 to ≤1.91	> 1.91
Animal protein	≤ 0.54	> 0.54 to ≤0.84	> 0.84 to ≤1.17	> 1.16
Plant protein	≤ 0.43	> 0.43 to ≤0.56	> 0.56 to ≤0.74	> 0.74
DQI-I (out of 94)	≤ 56	> 56 to ≤63	> 63 to ≤70	> 70
DASH (out of 9)	≤ 2.50	> 2.50 to ≤3.50	> 3.50 to ≤4.50	> 4.50
MIND (out of 9)	≤ 3.50	> 3.50 to ≤4.50	> 4.50 to ≤5.00	> 5.00
MDS (out of 9)	≤ 3	> 3 to ≤4	> 4 to ≤5	> 5
DII	≤ -1.67	> -1.67 to ≤-0.64	> -0.64 to ≤0.15	> 0.15
**Female participants with Moderate/severe sarcopenia**				
Total protein	≤ 1.00	> 1.00 to ≤1.28	> 1.28 to ≤1.68	> 1.68
Animal protein	≤ 0.47	> 0.47 to ≤0.65	> 0.65 to ≤0.89	> 0.89
Plant protein	≤ 0.44	> 0.44 to ≤0.58	> 0.58 to ≤0.80	> 0.80
DQI-I (out of 94)	≤ 59	> 59 to ≤67	> 67 to ≤73	> 73
DASH (out of 9)	≤ 3.50	> 3.50 to ≤4.00	> 4.00 to ≤5.00	> 5.00
MIND (out of 9)	≤ 4.00	> 4.00 to ≤4.50	> 4.50 to ≤5.50	> 5.50
MDS (out of 9)	≤ 3	> 3 to ≤4	> 4 to ≤5	> 5
DII	≤ -1.33	> -1.33 to ≤0.13	> 0.13 to ≤0.66	> 0.66

Protein intake is calculated in g per day/ kg body weight. Dietary patterns are measured using a total score for each pattern.
